# Clinical analysis of metastatic characteristics of infrapyloric lymph nodes (No.206) and terminal ileum lymph nodes in patients with right colon cancer

**DOI:** 10.1186/s12957-021-02414-z

**Published:** 2021-10-22

**Authors:** Jiangrui Liu, Yibin Su, Xing Liu, Jinfu Zhuang, Yuanfeng Yang, Guoxian Guan

**Affiliations:** 1 Department of Gastrointestinal Surgery, Quanzhou First Hospital affiliated to Fujian Medical University, No. 248 Dong Street, Licheng District, Quanzhou, 362000 Fujian China; 2 Department of Colorectal Surgery, the First Affiliated Hospital of Fujian Medical University, No. 20 Chazhong Road, Fuzhou, 350000 Fujian China

**Keywords:** Right colon cancer, Lymph node metastasis, No.206 group lymph node, Terminal ileum lymph node

## Abstract

**Background:**

D3 or complete mesocolic excision (CME) surgery has become a common surgical procedure for the treatment of colon cancer metastasis. Clinical misuse and overuse of lymph node dissection bring unnecessary burdens to patients. A detailed guidance for lymph node dissection in patients with T3 and T4 stage right colon cancer at different locations is urgently needed.

**Methods:**

A retrospective study was performed. Patients received D3 or CME surgery were divided into ileocecal group, ascending colon group, and hepatic flexure group according to the 9th edition of the Japanese Society for Cancer of the Colon and Rectum guidelines. The distributions of lymph node metastases were analyzed according to tumor infiltration depth (T stage) and tumor location.

**Results:**

The incidence of metastases in the paracolic area (or station), intermediate area, and main (or central) area was 38.4% (139/362), 12.7% (46/362), and 9.7% (35/362), respectively. The proportion of patients having No.206 and terminal ileum lymph nodes metastases was 7.7% (14/181) and 3.7% (9/244), respectively. No.206 lymph node metastasis is related to tumor location (*χ*^2^ = 7.955, *p* = 0.019) and degree of differentiation (*χ*^2^ = 18.99, *p* = 0.000), and terminal ileum lymph node metastasis is related to tumor location (*χ*^2^ = 6.273, *p* = 0.043). Patients with T3/T4 hepatic flexure cancer received radical right hemicolectomy in addition to No.206 lymph node dissection.

**Conclusion:**

Radical right hemicolectomy and No.206 group lymph node dissection are necessary for T3 and T4 stage colon cancer therapy.

## Background

Selection of a reasonable surgical strategy is directly related to the incidence of surgical complications, postoperative recovery, and 5-year survival rate of patients with advanced right colon cancer [1, 2]. The degree of lymph node metastasis of right colon cancer directly affects the efficacy of surgical treatment [3, 4]. The lymphatic vessels of the colon are accompanied by arteries, and the path starts from the peripheral lymphatic vessels in the intestinal wall to the lymph nodes adjacent to the tumor (paracolic area) and forms the reflux pathway along the mesenteric lymph nodes (intermediate area), the mesenteric root lymph nodes (main area), and the para-arterial lymph nodes at the margin of the longitudinal axis of the bowel (paracolic area + intermediate area) [5, 6]. Some data show that the most important prognostic factor is LN metastasis, so reasonable lymph node dissection is of great significance [7, 8].

At present, complete mesocolic excision (CME) could not only remove the lymph nodes beside the colon, but also further remove the lymph nodes that feed the roots of blood vessels, increasing the rate of lymph node acquisition [9, 10]. However, whether or not to routinely clean the lymph nodes at D3 radical resection for each colon cancer patient remains to be further explored [11]. The enlarged right colon cancer resection performed during the removal of the lymph nodes in the group 223 (No.223) lymph nodes and group 206 (No.206) lymph nodes increased the risk of injury to the superior mesenteric artery and vein, intraoperative bleeding, and postoperative chyle leakage [12, 13]. We need to find the best way to determine when to perform D2/D3 radical resection and when to remove NO.206 lymph nodes on D3 radical resection of colon cancer. At present, there are few analyses on the lymph node metastasis of terminal ileum, which is connected to the cecum, located in the lower right abdomen of the human body, and adjacent to the appendix. This article aims to explore the lymph node metastasis of right colon cancer, especially the metastasis in No.206 lymph nodes and the terminal ileum, in order to provide a reference for the scope of lymph node dissection and surgical methods.

## Methods

### Study population

This study was approved by the Ethics Committee of the First Affiliated Hospital of Fujian Medical University. A total of 390 patients diagnosed with right colon cancer and performed D3 or CME surgery by the same group of colorectal surgeons in the First Affiliated Hospital of Fujian Medical University from January 2010 to December 2017 were collected, and a total of 362 cases were screened. Inclusion criteria are as follows: (1) primary stage I, II, and III right colon cancer; (2) D3 radical resection or CME + R0 resection; and (3) the number of lymph nodes submitted for each case was 12 or more. Exclusion criteria are as follows: (1) patients with multiple primary colon cancer, familial adenomatous polyposis (FAP), other malignant tumors at the same time or in the past, recurrence or metastasis after colon cancer surgery and (2) neoadjuvant therapy before surgery.

### Intervention

Patients with T1 and T2 stages of right colon cancer were treated with D2 dissection, and patients with T3 and T4 stages were treated with D3 dissection. D3 dissection of the right colon for the treatment of ileocecal cancer included No.203 and No.213. D3 dissection and radical resection of the right colon for the treatment of ascending colon cancer and hepatic flexure colon cancer included No.203, No.213 and No.223. T3 + T4 stage hepatic flexure cancer of the colon was treated with radical right hemicolectomy, and the lymph nodes in group No.206 were included in the radical right hemicolectomy.

### Definition and pathological examination methods of lymph node detection

After the operation, the surgeon in the operation team dissected the gross specimen to find, separate, and locate the lymph nodes, a small part of which was obtained by the pathologist. All the obtained lymph nodes were immersed and fixed in formalin solution, and stained with standard HE, and were identified by 2–3 experienced pathologists.

The statistics and labeling of the colorectal lymph nodes and the classification of ileocecal group, ascending colon group, and hepatic flexure group were carried out in accordance with the 9th edition of the Japanese Society for Cancer of the Colon and Rectum guidelines [5]. The tumor infiltration depth (T stage) is classified according to the American Joint Committee on cancer (AJCC) method [14]. Endoscopic treatment was used for tumor staging under necessary conditions.

No.206 lymph nodes are lymph nodes distributed along the proximal end of the right gastroepiploic artery. No.206 lymph nodes are mainly distributed along the inferior pyloric artery down to the confluence of the right gastroepiploic vein and the superior anterior pancreaticoduodenal vein. Metastasis lymph nodes that appear in these areas were defined as #206 metastases. No.212 lymph nodes are intermediate lymph nodes in the right colic artery. Only these metastasis lymph nodes were defined as #212 metastases.

### Statistical analysis

SPSS 20.0 statistical software and chi-square test were used to perform statistical analysis, and *p* < 0.05 indicate an accepted statistical significance. The single factor analysis method (chi-square test) was initially applied. The chi-square segmentation method (*a*' = *a*/[*k*(*k* − 1)/2 + 1], *k* = number of groups) was used for pairwise comparisons. Small sample Fisher exact probability calculation method can be used. Fisher’s exact probability calculation method was used for small samples. Then, the significant related factors in the single factor analysis (*p* < 0.05) were screened for logistic multivariate regression analysis, with a = 0.05 as the significance level.

## Results

### Distribution of lymph node metastases caused by mesocolic lymphatic spread in right colon cancer

Three hundred sixty-two patients were included for the study, among whom 181 received dissection of the infrapyloric nodes. In addition, 244 of 362 patients received extended resection of the terminal ileum. Sixty-three patients received infrapyloric dissection as well as extended resection/dissection of the terminal ileum. The proportion of patients having lymph nodes metastases (*n* = 155) was 42.8%. The overall number of dissected lymph nodes was 11892, of which 619 (5.2%) were metastatic. The proportion of patients who possess lymph nodes metastases in the region paracolic area was 38.4% (139/362). Among them, the total number of lymph nodes was 1601, and the metastatic lymph nodes were 252, with a degree of metastasis of 15.7%. The incidence of metastases in the intermediate area was 12.7% (46/362), and 6.5% lymph nodes (34/525) were metastatic. The proportion of patients who possess lymph nodes metastases in the main area was 9.7% (35/362). Among them, the total number of dissected lymph nodes was 786, and the metastatic lymph nodes were 37, with a degree of metastasis of 4.7%. Accordingly, the incidence of metastases in the paracolic area, the intermediate area, and the main area was 38.4% (139/362), 12.7% (46/362), and 9.7% (35/362), respectively. The difference was statistically significant (*χ*^2^ = 111.6, *p* = 0.000, Table 1).

**Table 1:** Distribution of lymph node metastases in all patients

Total cases	Metastatic cases	Metastasis rate (%)	Lymph node metastasis rate (%) in different pathological N stage				
			N1	N2	N3	*χ*^2^	*P value*
362	155	42.8	38.4%	12.7%	9.7%	111.6	0.000
			(139/362)	(46/362)	(35/362)		

### Distribution of lymph node metastases by tumor location

As shown in Table 2, lymph node metastasis rate in the ileocecal group, ascending colon group, and hepatic flexure group was 46.7% (43/92), 43.7% (59/135), and 39.2% (53/135), respectively, and the difference was not statistically significant (*χ*^2^ = 1.319, *p* = 0.517). The distribution of lymph nodes metastases in proximal regions along the bowel (paracolic area) was 43.5% (40/92), 40% (54/135), and 33.3% (45/135), respectively, and the difference was not statistically significant (*χ*^2^ = 2.614, *p* = 0.271). No.222 and No.223 lymph node metastasis was not found in the ileocecal group, and no.202 and No.203 lymph node metastasis was not found in the hepatic flexure colon group.

**Table 2:** Distribution of lymph node metastases by tumor location

Tumor location	Total metastasis rate (% )	Metastasis rate (%) in paracolic area	Metastasis rate (%) in intermediate area						Metastasis rate (%) in main area			
			No.202	No.212	No.222	*χ*^2^	*p* value	No.202	No.212	No.222	*χ*^2^	*p* value
Ileocecal group	46.7%	43.5%	12.0%	2.2%	0	16.629	0.000	6.5%	2.2%	0	7.209	0.027
	(43/92)	(40/92)	(11/92)	(2/92)	(0/92)			(6/92)	(2/92)	(0/92)		
Ascending colon group	43.7%	40%	6.7%	3.0%	4.4%	0.415	0.375	2.2%	4.4%	3.0%	0.415	0.375
	(59/135)	(54/135)	(9/135)	(4/135)	(6/135)			(3/135)	(6/135)	(4/135)		
Hepatic flexure group	39.2%	33.3%	0	4.4%	6.7%	0.635	0.298	0	6.7%	5.9%	0.063	0.500
	(53/135)	(45/135)	(0/136)	(6/135)	(9/135)			(0/135)	(9/135)	(8/135)		
*χ*^2^	1.319	2.614	15.622	0.963	6.171	/	/	9.661	2.498	6.078	/	/
*P* value	0.517	0.271	0.000	0.618	0.046	/	/	0.008	0.287	0.048	/	/

In ileocecal group (Table 3), lymph node metastasis rate in No.202, No.212, and No.222 was 12.0%, 2.2%, and 0.0%, respectively. The difference between groups in intermediate area was statistically significant (*χ*^2^ = 16.629, *p* = 0.000). Among them, No.202 is significantly higher than No.222 (12.0% vs 0); the difference is statistically significant (*χ*^2^ = 11.699, *p* = 0.000). In addition, the lymph node metastasis rate in No.203, No.213, and No.223 was 6.5%, 2.2%, and 0.0%, respectively. The difference between groups in main area was statistically significant (*χ*^2^ = 6.202, *p* = 0.014). Our results suggest that in the ileocecal group, metastases dominated by the ileocolic, right colic, and middle colic arteries.

**Table 3:** Distribution of lymph node metastases in ileocecal group

Tumor location	Lymph node metastasis rate (%) in intermediate area				Lymph node metastasis rate (%) in main area			
	No.202 (%)	No.222 (%)	*χ*^2^	*P value*	No.203 (%)	No.223 (%)	*χ*^2^	*P value*
Ileocecal group	12.0%	0	11.699	0.000	6.5%	0	6.202	0.014
	11/92	0/92			6/92	0/92		

In the ascending colon group, lymph node metastasis rate in No.202, No.212, and No.222 was 6.7%, 3.0%, and 4.4%, respectively. The difference between groups in intermediate area region was not statistically significant (*χ*^2^ = 0.415, *p* = 0.375). In addition, the lymph node metastasis rate in No.203, No.213, and No.223 was 2.2%, 4.4%, and 3.0%, respectively. The difference between groups in main area was not statistically significant. Among them, No.202 is similar to No.212 (6.7% vs 3.0%), No.212 is similar to No.222 (3.0% vs 4.4%), No.203 is similar to No.213 (2.2% vs 4.4%), and No.213 is similar to No.223 (4.4% vs 6.7%). The difference is not statistically significant (*χ*^2^ = 2.020, *p* = 0.127; *χ*^2^ = 1.034, *p* = 0.250; *χ*^2^ = 0.415, *p* = 0.375; *χ*^2^ = 0.415, *p* = 0.375), indicating that cancer in the ascending colon group could metastasize to the ileocolic, right colic, and middle colic arteries

In the hepatic flexure colon group, lymph node metastasis rate in No.202, No.212, and No.222 was 0%, 4.4%, and 6.7%, respectively. The difference between groups in intermediate area was not statistically significant (*χ*^2^ = 0.635, *p* = 0.298). In addition, the lymph node metastasis rate in No.203, No.213, and No.223 was 0%, 6.7%, and 5.9%, respectively. The difference between groups in main area was not statistically significant (*χ*^2^ = 0.065, *p* = 0.500). Among them, No.212 is similar to No.222 (4.4% vs 6.7%), No.213 is similar to No.223 (6.7% vs 5.9%), and the difference is not statistically significant (*χ*^2^ = 0.635, *p* = 0.298; *χ*^2^ = 0.063, *p* = 0.500), indicating that cancer in the ascending colon group could metastasize to the ileocolic, right colic, and middle colic arteries.

### Mesocolic lymphatic spread by pathological T stage

According to the depth of tumor invasion, the total metastasis rate of right colon cancer in T1, T2, T3, and T4 were 11.1%, 14.3%, 42.2%, and 69.9%, respectively. There were statistical differences between groups (*χ*^2^ = 33.56, *p* = 0.000). Only N1 lymph node metastasis was found in both T1 and T2, which was 11.1% and 14.3% respectively. N1, N2, and N3 in T3 and T4 groups all had lymph node metastasis, with metastasis rates of 38.2%, 11.6%, and 7.9% and 63.9%, 26.5%, and 25.3%, respectively. We found that there were statistical differences in the metastasis rates of T1, T2, T3, and T4 at paracolic area, intermediate area, and main area (*χ*^2^ = 28.04, *p* = 0.000; *χ*^2^ = 17.21, *p* = 0.000; *χ*^2^ = 23.6, *p* = 0.000), indicating that the depth of invasion were associated with the rate of total lymph node metastasis in right colon cancer (Table 4).

**Table 4:** Distribution of lymph node metastases by pathological T stage

T stage	Total cases	Metastatic cases	Metastasis rate (%)	N1 (%)	N2 (%)	N3 (%)
T1	9	1	11.1	11.1 (1/9)	0 (0/9)	0 (0/9)
T2	21	3	14.3	14.3 (3/21)	0 (0/21)	0 (0/21)
T3	277	117	42.2	38.2 (106/277)	11.6 (32/277)	7.9 (22/277)
T4	83	58	69.9	63.9 (53/83)	26.5 (22/83)	25.3 (21/83)
*χ*^2^	/	/	33.56	28.04	17.21	23.6
*P* value	/	/	0.000	0.000	0.000	0.000

### Separate metastasis in No.206 and terminal ileum lymph nodes

The proportion of patients having No.206 lymph nodes metastases was 7.7% (14/181). The overall number of dissected No.206 lymph nodes was 432, of which 14 (3.2%) were metastatic. As shown in Table 5, No.206 lymph node metastasis is related to tumor location (*χ*^2^ = 7.955, *p* = 0.019) and degree of differentiation (*χ*^2^ = 18.99, *p* = 0.000).

**Table 5:** The metastasis in No.206 lymph nodes

Clinicopathological characteristics	Metastatic cases	Total cases	Metastasis rate (%)	*χ*^2^	*P* value
Age (years)				0.022	0.555
< 60	6	81	7.4		
≥ 60	8	100	8		
Gender				2.779	0.083
Male	5	103	4.9		
Female	9	78	11.5		
Tumor location				7.955	0.019
Ileocecal group	1	29	3.4		
Ascending colon group	1	62	1.6		
Hepatic flexure group	12	90	13.3		
Macroscopically type				3.361	0.186
Polypoid pattern	2	55	3.6		
Ulcerative pattern	9	108	8.3		
Infiltrative pattern	3	18	16.7		
Tumor size (cm)				0.112	0.497
< 5	4	59	6.8		
> 5	10	122	8.2		
Differentiation				18.99	0.000
High	0	17	0		
Moderate	8	146	5.5		
Low	6	18	33.3		
Microscopical types				2.759	0.090
Adenocarcinoma	7	126	26.9		
Mucous and signet ring cancer	7	55	12.7		
T stage				1.174	0.338
T1 + T2	0	13	0		
T3 + T4	14	168	8.3		

The proportion of patients having terminal ileum lymph nodes metastases was 3.7% (9/244). The overall number of dissected terminal ileum lymph nodes was 253, of which 17 (6.7%) were metastatic. As shown in Table 6, terminal ileum lymph node metastasis is related to tumor location (*χ*^2^ = 6.273, *p* = 0.043).

**Table 6:** The metastasis in terminal ileum lymph nodes

Clinicopathological characteristics	Metastatic cases	Total cases	Metastasis rate (%)	*χ*^2^	*P* value
Age (years)				0.330	0.416
< 60	3	104	2.9		
≥ 60	6	140	4.3		
Gender				0.411	0.814
Male	6	138	4.3		
Female	3	106	2.8		
Tumor location				6.273	0.043
Ileocecal group	5	65	7.7		
Ascending colon group	4	94	4.3		
Hepatic flexure group	0	85	0		
Macroscopically type				4.960	0.084
Polypoid pattern	0	65	0		
Ulcerative pattern	7	159	4.4		
Infiltrative pattern	2	20	10		
Tumor size (cm)				2.119	0.134
< 5	1	82	1.2		
> 5	8	162	4.9		
Differentiation				2.648	0.266
High	0	15	0		
Moderate	7	208	3.4		
Low	2	21	9.5		
Microscopical types				0.655	0.337
Adenocarcinoma	7	159	4.4		
Mucous and signet ring cancer	2	85	2.3		
T stage				1.778	0.203
T1 + T2	0	39	0		
T3 + T4	9	205	4.4		

## Discussion

The therapeutic effect of colon cancer is far from that of rectal cancer. The main reason is that the widespread implementation of total mesorectal excision is of great significance for reducing the recurrence rate and improving the survival rate of patients [15]. In 2009, Hohenberger first proposed CME [16], which referred to the theoretical basis of TME. In addition, numerous surgical experts reported on Japanese D3 radical resection [17, 18]. These two procedures are superior to some of the procedures recently reported in other countries, but they also illustrate the diversity of current colon tumor surgeries. Therefore, it is necessary to further standardize and optimize colon cancer surgery methods to further improve the clinical prognosis of colon cancer.

Long-term survival after colorectal cancer may be improved by more extensive resection of the primary tumor and lymph nodes [19, 20] Resection of the gastroepiploic and infrapyloric lymph nodes in the gastrocolic ligament has been proposed as a standard procedure when resecting tumors located in the proximity of the flexures or in the transverse colon [21]. In addition to the central direction, the lymph node drainage of the right colon cancer may also cause the subpyloric drainage [22]. Colon cancer of hepatic flexure is likely to have metastasis of the infrapyloric lymph nodes (No.206), which are not regional lymph nodes [23]. Lymph node dissection of No.206 group belongs to extended right hemicolectomy, which involves many vascular variations and complicated peripheral anatomical structure [22]. Previously, it was reported in the literature that the lymph node metastasis rate in the No.206 group of the right colon cancer was 1.5–2.6% [24]. The result of this study was 3.9% (14/362), which is slightly higher than similar studies. The lymph node metastasis rate of No.206 group in the ileocecal group, ascending colon group, and hepatic flexure colon group was 3.4% (1/29), 1.6% (1/62), and 13.3% (12/90), respectively (*χ*^2^ = 7.955, *p* = 0.019). The metastasis rate in the hepatic flexure colon group was significantly higher than that in the ascending colon group and the ileocecal group. In addition, the lymph node metastasis of No.206 group all occurred in T3 + T4 stage (Table 7). Therefore, radical right colon resection should be performed for hepatic flexure colon cancer in T3 and T4 stage.

**Table 7:** Literature review of lymph node metastasis in the terminal ileum of right colon cancer

	Toyota et al. (1995) [25]	Lan et al. (2011) [26]	Kang et al. (2018) [27]	Present study	Summary
Study design	Retrospective study	Retrospective study	Retrospective study	Retrospective study	
Total patients	275	103	752	244	1374
Tumor location	C/A/T (87/188/53)	C/A (47/56)	C/A/HF (82/554/116)	C/A/HF (965/94/85)	C/A/HF or T (281/892/254)
Metastasis rate in the terminal ileum	2.9% (8/275) NA	6.8%(7/103) C:10.6%(5/47) A:3.6% (2/56)	2.7%(20/752) C:7.3%(6/82) A:2.2%(12/554) HF:1.7%(2/116)	3.7%(9/244) C:7.7%(5/65) A:4.3%(4/94) HF:0(0/85)	3.2%(44/1374) C:8.2%(16/194) A:2.6%(18/704) HF: 1% (2/201)
Pathological stage	NA	III: 0 (0/33) IV:26.0% (7/27)	III:1.1% (3/262) IV:15.5%(17/110)	III:3.7% (9/244) IV:NA	III: 2% (12/593) IV:17.5%(24/137)

*A* Ascending colon group, *C* Ileocecal group, *HF* Hepatic flexure group, *NA* Not available, *T* Transverse colon group

In this paper, the right colon tumors are grouped according to the position of the tumor center, which was identified with histological examination or endoscopy by experts. Our results showed that the metastasis in the ileocecal group was dominated by the ileocolic, right colic, and middle colic arteries, and the middle colic artery metastasis was rare. Therefore, for the radical resection of ileocecal cancer, NO.222 and NO.223 lymph nodes are not necessary to be routinely removed. Other guidelines do not support a dissection of the middle colic artery at its origin in case of ascending colon cancer. However, it was observed that metastasis of the hepatic flexure colon group and the ascending colon group all spread back through the in this study. This study shows that both hepatic flexure colon group cancer and ascending colon group should be treated with D3 radical right hemicolectomy including remove of NO.222 and NO.223 lymph nodes.

For the distribution of lymph node metastases by tumor location, we found that the lymph node metastasis rates of the paracolic area (or station), intermediate area, and main (or central) area were 38.4%, 12.7%, and 9.7%, respectively. It suggested that the metastatic ability of cancer cells was significantly correlated with the distance between the lymph nodes (*p* < 0.05). Our data show that the lymph node metastasis in right colon cancer patients first appear in the bowel axis lymph nodes, and then to the central lymph nodes. Colon tumor cells tend to metastasize to lymph nodes close to the axis of the bowel [28, 29]. In addition, the total metastasis rate of right colon cancer in T1, T2, T3, and T4 were 11.1%, 14.3%, 42.2%, and 69.9%, respectively. Consistent with the results of previous studies, these results also suggest that the T stage was correlated with the lymph node metastasis rate of right colon cancer (*χ*^2^ = 29.6555, *p* = 0.000). With the deepening of colon cancer infiltration depth, the metastasis rate of bowel axis and central lymph node increased significantly, similar to domestic and foreign studies [30]. However, we have to admit that we may have some deviations in the lymph node collection process. Due to the limitations of resources and technology, we used the manual method to collect lymph nodes. In subsequent studies, we will try to use new lymph node collection techniques such as lymph node revealing solutions containing glacial acetic acid, ethanol, water, and formalin (GEWF) to improve the harvesting rates of lymph nodes [31, 32].

The vascular anatomy of the right colon has showed several significant changes, including the absence of some branches (lack of RCA in 27.4% of cases), extra branches, common trunk, and posterior superior mesenteric vein routes [33]. Therefore, most studies indicate that the proximal ileum resection margin for right colon cancer should be 5–10 cm from the tumor margin [34]. In this study, we have demonstrated that terminal ileum lymph node metastasis is related to tumor location (*χ*^2^ = 6.273, *p* = 0.043). The research progress shows that lymph node metastasis follows the supporting blood vessels. The distal ileum is the ileum fed by the branches of the ileocolonic artery, and it is also a possible area for lymphatic metastasis of right colon cancer [35]. The distal ileum is also important in physiological functions, because the terminal ileum absorbs combined bile acids and absorbs vitamin B12 [36]. However, there are relatively limited studies on ileal lymph node metastasis, and only a few reports describe partial ileum lymph node metastasis. In this study, we reported that the proportion of patients having terminal ileum lymph nodes metastases was 3.7% (9/244). The overall number of dissected terminal ileum lymph nodes was 253, of which 17 (6.7%) were metastatic. Lan et al. retrospectively analyzed 103 patients with right colon cancer and pointed out that terminal ileum lymph node metastasis only exists in stage IV patients [26]. Kang SI et al. retrospectively analyzed 752 patients, focusing on the impact of tumor location and pathological staging on lymph node metastasis in the terminal ileum [27]. In this study, peri-ileal lymph nodes were detected in 244 of 362 patients, of which 3.7% (9/244) were metastatic. In the ileocecal group, 7.7% (5/65) of patients have metastasized to peri-ileal lymph nodes. The reason for the low metastasis rate of peri-ileal lymph nodes may be the resistance of the abundant lymphatic tissue in the terminal ileum to tumor metastasis [27]. Therefore, in most cases, extensive resection of the lymphatic system of the terminal ileum is not necessary. In this study, the metastasis rate of peri-ileal lymph nodes was detected in patients with TNM stage III, and the metastasis rate was 3.7% (9/244). Based on the above research results, the total metastasis rate of peri-ileal lymph nodes of right colon cancer was 3.2% (8.2% in the ileocecal group, 2.6% in the ascending colon group, and 1% in the hepatic flexure colon group). The “10 cm principle” of ileectomy is suitable for preventing local recurrence in patients with advanced cecal cancer.

## Conclusion

In conclusion, we demonstrated that radical right hemicolectomy and dissection of No.206 lymph nodes are necessary for T3 and T4 stage colon cancer. The dissection of peri-ileal LNs should be prioritized to the preservation of function. We also found that T2/T1 right colon cancer should be treated with D2 radical resection: T3/T4 ileocecal cancer should undergo D3 radical resection and be removed together with No.203 and No.213 lymph nodes. We hope that our research could provide a certain basis for the implementation of lymph node dissection for colon cancer patients in different periods.

## Data Availability

Data is available from the authors by request.

## Abbreviations

CME: Complete mesocolic excision
T stage: Tumor infiltration depth
No.223: Lymph nodes in the group 223
No.206: Lymph nodes and group 206
FAP: Familial adenomatous polyposis
AJCC: American Joint Committee on cancer
